# Hepatitis Virus and Hepatocellular Carcinoma: Recent Advances

**DOI:** 10.3390/cancers15020533

**Published:** 2023-01-15

**Authors:** Chen Shen, Xin Jiang, Mei Li, Yao Luo

**Affiliations:** Department of Laboratory Medicine, West China Hospital, Sichuan University, Chengdu 610041, China

**Keywords:** hepatocellular carcinoma, hepatitis virus, molecular mechanism, diagnosis, treatment

## Abstract

**Simple Summary:**

Hepatocellular carcinoma (HCC) is a global health challenge. Hepatitis virus infection (including HBV, HCV and HDV) is one of the major risk factors for HCC development. These viruses induce hepatocyte cancer by causing chronic hepatitis and by a variety of complex mechanisms. Here we discuss the mechanisms by which several hepatitis viruses induce HCC, as well as new diagnostic and therapeutic approaches to HCC based on the findings of these mechanisms. Finally, we also discuss the potential relationship between HEV and HCC.

**Abstract:**

Hepatocellular carcinoma (HCC) remains a global health challenge, causing 600,000 deaths each year. Infectious factors, including hepatitis B virus (HBV), hepatitis C virus (HCV) and hepatitis D virus (HDV), have long been considered the major risk factors for the development and progression of HCC. These pathogens induce hepatocyte transformation through a variety of mechanisms, including insertional mutations caused by viral gene integration, epigenetic changes, and the induction of long-term immune dysfunction. The discovery of these mechanisms, while advancing our understanding of the disease, also provides targets for new diagnostic and therapeutic approaches. In addition, the discovery and research of chronic HEV infection over the past decade indicate that this common hepatitis virus also seems to have the potential to induce HCC. In this review, we provide an overview of recent studies on the link between hepatitis virus and HCC, as well as new diagnostic and therapeutic approaches to HCC based on these findings. Finally, we also discuss the potential relationship between HEV and HCC. In conclusion, these associations will further optimize the diagnosis and treatment of infection-associated HCC and call for better management policies.

## 1. Introduction

Liver cancer remains a global health challenge, with an increasing incidence worldwide. It is estimated that 1 million people will be affected by liver cancer each year by 2025 [1]. Hepatocellular carcinoma (HCC) is the most common form of liver cancer, accounting for about 90% of all cases [2]. HCC is the fifth most common cancer and the second most common cause of cancer death [3,4]. Every year, there are more than 700,000 new cases of HCC worldwide, and more than 600,000 people die of HCC, among which the incidence in males is higher than that in females [5]. HCC is mainly distributed in China, Southeast Asian countries and sub-Saharan Africa, with the highest incidence of HCC in east Asia and southeast Asia, and most cases occur in the age range of 30–60 years [5,6]. In China, liver cancer is an important factor affecting people’s health, ranking second among six major cancers (lung/bronchial, liver, stomach, esophagus, colorectal and pancreatic). A study using raw data from China’s National Mortality Surveillance System to assess mortality rates for all cancers and site-specific cancers from 2004 to 2018 showed that liver cancer was an important cause of death in people < 65 years old in China, accounting for 44.35% of all cancer deaths, and HCC accounted for the vast majority of these liver cancer cases [7].

The main risk factors for HCC include liver cirrhosis, chronic hepatitis B virus (HBV) infection, hepatitis C virus (HCV) infection, hepatitis B virus and hepatitis D virus (HDV) co-infection or overlapping infection, long-term consumption of aflatoxin-contaminated food, long-term alcohol consumption, obesity, smoking and type II diabetes mellitus [2,3,5]. Infectious factors, including hepatitis B virus (HBV), hepatitis C virus (HCV) and hepatitis D virus (HDV), play a very important role in the pathogenesis of HCC, although the incidence of HCC caused by non-infectious factors is increasing. HBV infection is the most important risk factor for HCC, accounting for about 50% of the incidence of HCC [2,8]. The risk of HCV infection leading to HCC is greatly reduced by a sustained virology response (SVR) with direct-acting antiviral agents (DAAs), but approximately 30% of HCC is due to HCV infection [2,4,5,9]. Co-infection or overlapping infection of HDV and HBV will increase the risk of HCC in patients with chronic HBV infection by two- to six-fold and accelerate the progression of HCC [5]. There are significant differences in the risk factors for HCC in different regions. In sub-Saharan Africa and most Asian countries, except Japan, HBV infection is a common factor for HCC, while HCV infection is a major infectious factor for HCC in some other regions [6,10]. Overall, the Asia–Pacific region has the highest rates of HBV and HCV infection in the world, with 74% of global liver cancer deaths occurring in Asia [11].

The pathological mechanism of HCC is a complex multi-step process. The interaction of various factors leads to malignant transformation of hepatocytes and early development of HCC. These include genetic predisposition, viral and non-viral risk factors and their interactions, the cellular microenvironment composed of various immune cell involvement, and the severity of the underlying chronic liver diseases. More than 90% of HCC cases occur in patients with chronic liver disease [12,13]. Most patients with acute HBV or HCV infection will not develop HCC, and the long-term host immune response and chronic inflammation caused by the failure of acute infection clearance may be the key determinants of the progression from infection to severe liver diseases such as cirrhosis or HCC [8]. Chronic hepatitis caused by chronic viral infection and continuous immune response leads to liver tissue damage, and further causes liver fibrosis and cirrhosis. Cirrhosis is a susceptibility factor for HCC, while advanced liver fibrosis is highly associated with HCC risk [4,14]. In the process of the development of chronic liver disease and cirrhosis of the liver, components of the immune system such as the microenvironment and the function of the virus directly under the influence of liver cells gradually accumulate a large amount of DNA damage, chromosomal instability, new blood vessels in the early generation and epigenetic change, causing liver cell transformation, which is the basis of most HCC incidence [2,15].

**Figure 1:**
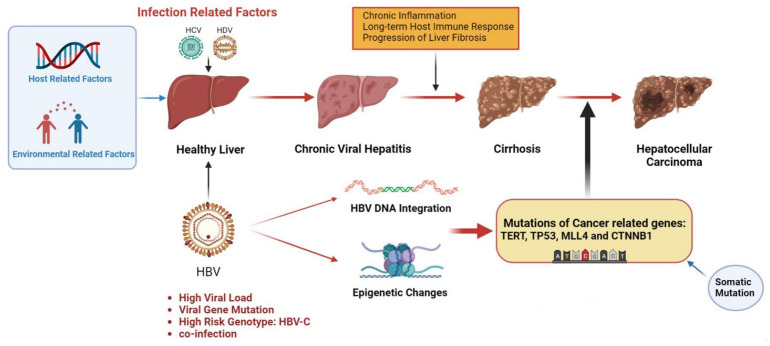
Pathogenesis of infection-induced hepatocellular carcinoma. Infectious factors include HBV, HCV and HDV, which mainly mediate chronic inflammation and persistent immune response through chronic viral hepatitis, leading to liver fibrosis and cirrhosis, inducing somatic mutations, and eventually leading to HCC. HBV infection is a major infectious factor with a unique oncogenic mechanism. HBV can cause cancer-related gene mutations through gene integration and epigenetic changes and promote the occurrence of HCC. Other virus-related risk factors include high viral load, viral gene mutations and high-risk genotypes. Environmental and host-related factors play a synergistic role. The figure material is referenced from Biorender.

HBV, HCV and HDV cooperatively infect cells through various mechanisms, leading to the occurrence of HCC (Figure 1). Common mechanisms among the three viruses include (1) persistent hepatitis and immune-mediated oxidative stress damage caused by chronic viral infection; (2) intracellular oxidative stress damage induced by viral proteins; (3) the abnormal regulation of cell signaling pathways by viral proteins (such as HBx, L-HDAg, S-HDAg, HCV core, NS3 and NS5A/B) [16]. As a hepatophilic DNA virus, HBV can integrate its viral DNA into the host genome and lead to cell carcinogenesis through insertional mutagenesis or the expression of viral proteins (such as HBx) [3,16,17]. Chronic hepatitis C (CHC) infection promotes metabolic reprogramming leading to steatosis, which triggers hepatitis and stimulates the development of liver fibrosis and progression to cirrhosis, and most HCV-associated HCC is based on liver fibrosis or cirrhosis [18,19,20]. HDV as a defective virus must depend on HBV to survive. Acute HDV infection can lead to more serious liver diseases through co-infection or overlapping with HBV infection, while chronic HDV infection will increase the progression rate of liver fibrosis, accelerate the progression of patients to cirrhosis, and ultimately increase the risk of HCC [21,22].

Although the vast majority of HCC is induced by chronic liver disease, further studies have found that HBV-related HCC occurs in cirrhosis and the normal liver, indicating that viral infection may have its own unique molecular mechanism of carcinogenesis in addition to inducing chronic liver inflammation [17]. Among these molecular mechanisms, viral infection induces gene mutations in liver cells and triggers hepatocyte transformation, which is closely related to the occurrence of HCC. It has been found that approximately 25% of HCC tumors have inducible mutations. Although the prevalence of most of these mutations is low (10%), there are still some gene mutations that frequently occur in the hepatocytes of HCC patients, such as *TERT*, *TP53*, and *CTNNB1*. These genes are often tumor suppressor genes or proto-oncogenes in the host genome, which are closely related to the occurrence of hepatocellular carcinogenesis [23,24]. In addition to gene mutations, infectious diseases can also affect host gene expression through epigenetic regulation, leading to abnormalities in a variety of signaling pathways. Although these mechanisms driving HCC are random, some specific genetic abnormalities or epigenetic regulations are inextricably linked to HCC [25]. Thanks to the development of next-generation high-throughput sequencing technology, bio-information technology and proteomic analysis technology, we can understand the molecular mechanism of HCC from the perspectives of the genome, epigenetics, the transcriptome and proteome, and explore the gene expression patterns and epigenetic characteristics of tumor tissues and cells [3]. The discovery of novel mechanisms of viral carcinogenesis is critical, as these findings may ultimately lead to the development of vaccination strategies and HCC treatment decisions, leading to personalized medicine to reduce virus-mediated cancer mortality and improve the prognosis of HCC patients [2,26]. 

Among the five hepatitis viruses, hepatitis A virus (HAV) and hepatitis E virus (HEV) are generally considered only to cause acute hepatitis, so it is difficult to associate them with HCC [27,28,29]. However, an unexpected chronic HEV infection (persistent viremia over 3–6 months and excluding HBV co-infection) in a transplant patient was reported in 2008, and chronic HEV infection in immunodeficient patients has been reported in subsequent years [30,31,32]. Once HEV has been found to cause chronic infection, it is suggested that it has the potential to induce HCC similar to other chronic viral hepatitis viruses (HBV and HCV), as mentioned above. This potential of HEV has attracted the interest of a number of researchers, and some case studies of HEV’s association with HCC have been conducted worldwide [33,34]. However, these findings cannot fully and accurately explain whether HEV can lead to HCC, and these cases are often interfered with HBV co-infection and the patient’s immune deficiency. Further studies and discussions will demonstrate the value of monitoring HEV infection in HCC patients and the need to prevent HCC in HEV patients. To be sure, HEV surveillance (including serological examination and nucleic acid detection) is beneficial for immunodeficient patients.

In this review, we focus on the pathological mechanisms, diagnosis, and treatment of HCC caused by hepatitis virus. We focus on the role of HBV, HCV and HDV in the development of HCC, and discuss the potential diagnostic and therapeutic value based on these molecular mechanisms. Finally, we focus on the potential of HEV to cause chronic viral hepatitis and the possible association between chronic HEV infection and HCC.

## 2. Hepatitis-B-Virus-Induced Hepatocellular Carcinoma

Hepatitis B virus (HBV) infection remains an important global public health problem with significant morbidity and mortality [26,35,36]. Despite the availability of preventive vaccines and antiviral therapy to halt disease progression and reduce the risk of liver cancer, about 257 million people worldwide are still living with chronic hepatitis B virus infection. HBV causes more than 850,000 deaths annually and is the most common cause of HCC (44% to 55%) [5,35,36]. The majority of HBV cases occur in Asia and sub-Saharan Africa, where HBV infection accounts for 60% of HCC cases, compared with only 20% in the west [36,37,38]. HCC is also an important cause of death in patients with HBV. Compared with non-cirrhotic patients, HBV-related cirrhotic patients have a 31-fold increased risk of HCC and 44-fold increased mortality [5]. Several other risk factors for HCC that increase the risk of HCC in HBV carriers include the male sex, older age, family history, viral type, and cirrhosis. In Africa, HBV-infected patients develop HCC, possibly because of exposure to aflatoxin B1, which acts synergistically with HBV and increases the risk of hepatocellular carcinogenesis [2,5].

At present, the improvement of social health conditions, hepatitis B vaccination program and effective antiviral treatment have reduced the HBV infection rate in many areas with a high prevalence of hepatitis B [39,40]. However, even with universal vaccination programs, it is not intrinsically possible to prevent acute cases of HBV infection, especially in high-risk groups. The existing antiviral treatment for HBV cannot eliminate cccDNA in liver tissue, so as to achieve a complete cure of HBV. Population movements are also currently changing the prevalence and incidence in some low-prevalence countries, as these migrants often come from areas with high HBV carrier rates [36]. In addition, the incorrect use of blood transfusions and blood products, unsafe sex, intravenous drug use and mother-to-child transmission can all contribute to human transmission of HBV. 

HBV is a small enveloped hepatotropic DNA virus that replicates by reverse transcription [41]. HBV particles enter hepatocytes through the capture of liver-specific surface receptor solute carrier family 10 member 1 (SLC10A1) and Na^+^-taurocholate cotransporting polypeptide (NTCP) [42]. HBV contains relaxed circular double-stranded DNA (rcDNA), which is repaired to covalently closed circular DNA (cccDNA) by DNA repair enzymes after invasion of the host nucleus. cccDNA can be stably retained in the nucleus for a long time and functions as a small chromosome, which is the template for all viral mRNAs (such as pregenomic RNA (pgRNA)) and determines the persistence of HBV infection. In the cytoplasm, these viral mRNAs encode structural and regulatory proteins that are packaged into the viral capsid along with pgRNA, which is retrotranscribed with the viral polymerase to form rcDNA. Nucleocapsids containing HBV DNA are encapsulated by the host membrane, which is covered with all three forms of HBsAg, and then secreted from the cell as virions by multivesicular bodies [41,43,44]. Because of the persistence of cccDNA in the nucleus, HBV carriers are at risk of developing chronic HBV infection. With the progression of chronic HBV infection, the risk of end-stage liver disease, including cirrhosis and HCC, increases [15].

The development of HBV-induced HCC is a complex, multifactorial, and progressive process involving the interaction of the virus with endogenous mutagens and the host immune response to the virus [3,17,45]. HBV-associated HCC may also occur in non-cirrhotic livers, suggesting that in addition to stimulating host immune responses and driving chronic necrotizing inflammation in the liver, HBV also plays a direct role in liver transformation by triggering common and cause-specific oncogenic pathways [45,46]. With the development of next-generation sequencing technology and bio-information analysis technology, the molecular mechanism of HBV-induced HCC has been better understood, including key gene mutations caused by gene integration, chromosomal aberrations and epigenetic changes, and the dysregulation of cell signaling pathways caused by these mechanisms together. These signaling pathways play a crucial role in the benign-to-malignant transition of HCC [15,47]. Among these mechanisms, HBV DNA gene integration is a key step in HBV-induced HCC pathogenesis, which can regulate cellular gene expression [48]. The integration of the viral genome into the host genome can occur randomly, and although it is not necessary for viral replication, it is one of the important mechanisms of hepatocyte transformation [36]. Long-term and chronic HBV infection greatly increases the probability of viral gene integration and promotes carcinogenesis. Such integration events have been demonstrated in 75–90% of HCC tissues, and these integrations may further lead to the development of host–virus fusion transcripts [26,49]. If gene integration occurs in some key gene regions, then it may lead to the activation of some proto-oncogenes or the suppression of tumor suppressor genes. In addition, certain viral genotypes and HBV variants are also known to increase the risk of HCC. Epidemiological studies have shown that HBV genotypes C, D, and F have a higher lifetime risk of HCC development than genotypes A and B [14,36,50]. In this section, we focus on the mechanisms of HBV-induced HCC and the potential diagnostic and therapeutic value of these mechanisms.

### 2.1. HBV DNA Integration

HBV is a small 3.2 kb DNA virus which can promote cellular transformation by genomic integration [3,17]. Although HBV uses reverse transcription for replication, unlike retroviruses, integration is not a key step in the viral life cycle and does not produce replicative viruses [51]. This integration tends to occur randomly, with partial double-stranded rcDNA formed 90% of the time during the reverse transcription of pgRNA. As the genetic material of HBV, rcDNA can be used to supplement the cccDNA library and generate live virions that can continue to infect new hepatocytes [52]. For the remaining 10% of cases, the reverse transcription process does not produce rcDNA but synthesizes double-stranded linear DNA (dslDNA). dslDNA can exist in virus particles or be integrated into host genes [52,53].

Although HBV DNA integration is random, it is closely related to the occurrence of HCC, and about 75–90% of HBV-related HCC cases have reported HBV DNA integration [14,49]. In contrast, HBV integration events are less frequently detected in adjacent non-tumor tissues [54]. In a study of 177 HBV-related HCC patients, HBV integration was found in 88% of patients at the HBV/human junction site [17]. The frequent discovery of HBV gene integration in HCC patients highlights the potential importance of this mechanism in the HBV induction of HCC. Among them, HBx and HBsAg are genes with the highest degree of integration, both of which are important for the transmission of viral replication/infection [1,55]. HBV DNA integration induces HCC mainly through three mechanisms: (1) the chromosomal instability of HBV integrated DNA; (2) changing the expression or function of proto-oncogenes and tumor suppressor genes to drive the occurrence of liver cancer; (3) the expression of integrated mutant HBV protein [52,56].

Almost half of all HCC cases are associated with hepatitis B virus (HBV) infection, and long-term chronic infection can greatly increase the probability of viral gene integration and promote carcinogenesis. Therefore, the accurate identification of HBV integration sites at the single-nucleotide resolution and understanding of the mechanism of cell carcinogenesis induced by HBV DNA integration are crucial for a better understanding of the HCC cancer genome landscape and the disease itself [26]. The application of next-generation sequencing technology and bio-information technology has advanced our understanding of HBV gene integration.

**Figure 2:**
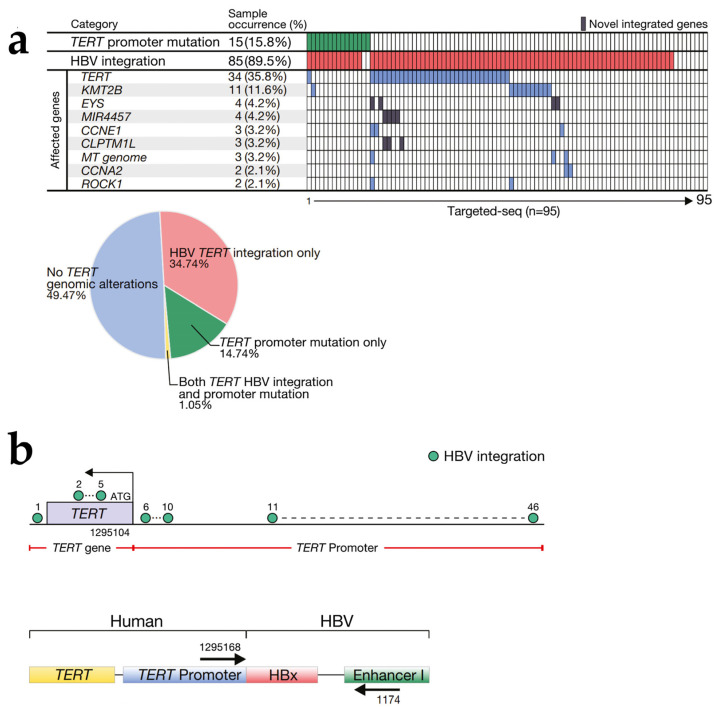
HBV DNA integration plays a very important role in HBV-induced HCC pathogenesis and can lead to insertional mutations in cancer-related genes. Among them, the most common site of HBV-mediated insertional mutation is located in the *TERT* promoter. (**a**) Common HBV DNA integration sites in HCC patients. The pie chart shows the distribution of *TERT* genomic alterations detected in the HCC cohort; (**b**) Schematic illustration showing frequent *TERT* HBV integration and its genomic location in human HCC tissues. After HBV integration in human HCC, the direct linkage of HBV *EnhI* and HBx DNA sequences to the human *TERT* promoter leads to *TERT* activation and telomerase overexpression, promoting cancer development. Reprinted with permission from Ref. [42]. 2020, John Wiley and Sons.

In a study of 177 HCC patients, investigators developed a viral-capture-based assay pipeline to analyze HBV integration (Figure 2). It was found that HBV integration in the human genome followed a different distribution in tumors compared to non-tumor tissues and tended to be enriched around cancer driver genes. Among them, three genes were repeatedly found to undergo HBV DNA integration in HCC tumor cells: *TERT* (*n* = 48), *CCNE1* (*n* = 4), and *KMT2B* (*n* = 3) [17]. In addition, further analysis revealed that the amount of HBV integration in the tumor was the only marker of the viral characteristics associated with a poor prognosis [17]. In another study, a novel strategy and bioinformatics platform, the Viral Integration Invoker (VIcaller), was used to identify clonal viral integrations in the human genome and provide estimated integration allele frequencies. It was found that *TERT* gene expression levels were significantly up-regulated in most samples with HBV integration [26]. The HBV integrations in the *TERT*, *MLL4* and *CCNE1* genes had the highest integration allele scores in the analysis of integration allele fragments. In addition, HBV integration with high-integration alleles was also found in seven genes, most of which have been reported to be associated with cancer, including *GAS7*, *SPECC1 (NSP)*, *RSPO2*, *NRG1*, *PRDM16*, *ARID1B*, and *AFF1*. In contrast, the total number of HBV integration events and integration allele scores were lower in non-tumor tissues [26].

The most common site of HBV-mediated insertional mutations is within the *TERT* promoter (15–25%), resulting in the overexpression of telomerase, which is responsible for the maintenance of telomere length, inhibition of cellular senescence, and promotion of cancer cell growth [2,57,58]. In a study of 95 HCC patients, researchers analyzed the distribution of common HBV DNA integration sites using targeted sequencing and found frequent HBV integration in *TERT*, along with increased *TERT* mRNA expression, which was associated with more aggressive tumor behavior [42]. The study also obtained the distribution of different forms of *TERT* genomic alterations, suggesting that the frequent occurrence of *TERT* gene changes in HCC patients is an important factor leading to carcinogenesis (Figure 2a) [42]. Through the study of *TERT* integration sites, HBV DNA integration at the *TERT* promoter was found to be the key to *TERT* gene overexpression. The further construction of different luciferase reporter genes revealed that HBV *EnhI* was the key viral component leading to *TERT* activation after *TERT* promoter integration, and the localization of HBV integration on the *TERT* promoter also regulated the degree of *TERT* transcriptional activation (Figure 2b) [42]. The array-based functional screening of siRNA libraries revealed that ETS transcription factor 4 (*ELF4*) played an important role in bridging the interaction between the *EnhI* and *TRET* genes. *ELF4* can bind to chimeric HBV *EnhI* at the *TERT* promoter, activate telomerase, and regulate HBV gene transcription [42]. *ELF4* is a DNA-binding transcription factor widely expressed in various tissues, including non-tumor liver diseases and liver cancer. The knockdown of *ELF4* can inhibit *TERT* activation in HCC tumor cells, providing a new therapeutic target [42,59].

In addition to modulating cancer-related genes, HBV DNA integration may also lead to functional virus–human transcript fusions [3,26]. For example, after HBV DNA is integrated into the *LINE* gene, the *HBV-LINE1* fusion transcript is generated, and its product activates Wnt/β-catenin signaling [60]. The formation of fusion transcript is closely related to the upper and lower breakpoints of HBV DNA integration and the direction of integration. VIcaller was used to identify HBV DNA integration sites and fusion transcripts, and researchers found that HBV integration in the *TERT* promoter region tended to be in the same direction as *TERT* [26]. The formation of these fusion transcripts is potentially related to the development of HCC by activating certain pathways associated with cell transformation (such as the Wnt pathway) or promoting telomerase overexpression. HBV DNA integration plays a very important role in the progression of HCC. The use of gene sequencing or nucleic acid amplification technology has potential diagnostic value for the detection of integrated viral genes, and specific integration sites can also be used as new targets for targeted therapy. However, obtaining liver tumor cells by puncture has certain risks, and non-invasive body fluid detection is more promising. Circulating cell-free DNA (cfDNA) has proven to be a powerful non-invasive blood-derived biomarker for early cancer detection, monitoring cancer development, and predicting cancer prognosis [61,62]. In healthy people, plasma cfDNA mainly comes from hematopoietic cells [63]. However, in cancer patients, it can also be released during tumor cell apoptosis or necrosis, which is called circulating tumor DNA (ctDNA). cfDNA has been shown to carry genetic and epigenetic information of tumor cells, which is of great value in the diagnosis and prognosis of HCC [64,65]. In one study, researchers developed a method based on cyclic single molecule amplification and resequencing (cSMART) to simultaneously detect the integration of viral DNA into the host genome and HBV mutations in cfDNA. This method maintains high sensitivity while being cheaper than whole-genome sequencing [14]. Through the study of HBV gene integration and mutation in HCC, a diagnostic model was established combined with machine learning. The diagnostic ability of this diagnostic model combined with AFP was better than that of AFP alone, and the diagnostic efficacy was improved from AUC = 0.78 to 0.88 [14]. 

### 2.2. Epigenetic Changes

Epigenetic changes alter gene expression, which affects cell and tissue phenotypes. Common epigenetic modifications include DNA methylation, chromatin covalent modifications, nucleosome position changes, mRNA modifications, and changes in the levels of microRNAs and lncRNAs. Epigenetic modification and genetic changes can synergistically promote tumor occurrence, progression and metastasis [3]. HBV-related epigenetic changes regulate abnormal gene expression and affect signaling pathways, although they do not alter gene composition. These changes will promote the occurrence of cell canceration and increase the possibility of HCC. 

In HBV-mediated epigenetic changes, chemical modifications of mRNA directly affect cellular transcription, translation and gene expression. Cellular RNA chemical modification can regulate RNA stability and turnover. Among several known RNA chemical modifications, n6-methyladenosine modification (m^6^A) is the most common internal mRNA modification in eukaryotic cells, which is usually enriched in the 3′-untranslated region (UTR) and around the stop codon of cellular mRNA [66,67]. Studies have found that HBV RNA transcripts are also modified by m^6^A, which has been shown to affect the life cycle and pathogenesis of HBV. However, HBV can change the expression of host genes by regulating the m^6^A modification of homologous RNA in host cells, so as to increase or decrease gene expression [68]. When the HBV-mediated modification of m^6^A affects the expression of cancer-related genes, cells become cancerous. Among the HBV-induced mRNA modifications, *PTEN* m^6^A modification is an example. *PTEN* is widely recognized as a tumor suppressor. *PTEN* is a metabolic regulator as well as a negative regulator of cell growth signaling pathways and is also involved in the regulation of innate immune responses activated by viral infection [69,70,71]. The levels of *PTEN* mRNA and m^6^A-modified *PTEN* mRNA in human liver biopsy specimens from healthy individuals, HBV-negative HCC patients, and HBV-positive HCC patients were compared and analyzed [72]. The results showed that HBV significantly increased the m^6^A modification of *PTEN* mRNA in cells (a two-fold increase), and m^6^A modification of *PTEN* mRNA negatively regulated its RNA and protein expression levels, led to the instability of *PTEN* mRNA and blocked the interferon signaling pathway. This change weakens the cancer suppressive effect of *PTEN*, which is conducive to the occurrence of HCC and immune escape [72]. In addition, HBV may also promote the development of HCC by activating the PI3K/AKT pathway and reducing the stability of *PTEN* mRNA [72].

Some members of non-coding RNAs (e.g., lncRNAs, microRNAs) are important components of epigenetic modifications and are involved in the regulation of gene expression, although they are not transcribed to form proteins. lncRNAs (long non-coding RNAs) are composed of 200–300 nucleotides and regulate gene expression through various mechanisms, including the recruitment of chromatin-modifying enzymes or the interaction with proteins to direct their binding to DNA [73,74]. Many lncRNAs have abnormal expression levels in tumor cells of HCC patients, many of which are involved in the regulation of cell adhesion, immune response and metabolism, and promote tumor progression [3,35,73,75]. The main function of the HBV HBx protein is to establish and maintain a cccDNA microchromosome with transcriptional activity. However, in addition to viral chromatin, HBx can recruit and regulate a variety of coding genes and non-coding RNA promoters [76]. In cell models related to HBV replication, researchers have found that HBx binds to the promoter region of *DLEU2*, enhances the transcription of *DLEU2*, and induces the accumulation of *DLEU2* RNA in infected hepatocytes [35]. *DLEU2* is a long non-coding RNA (lncRNA) expressed in the liver and increased in human HCC, which plays a role in the regulation of host target genes and HBV cccDNA [35]. This finding further demonstrated the epigenetic control of host genes by HBV and the transforming effect of HBx on liver cells in HBV-induced HCC [35]. MicroRNAs are short, consisting of 20 to 22 nucleotides resulting in non-coding RNAs that pair with the complementary 3′-untranslated region of messenger RNA, inhibiting its translation or causing its degradation. A single microRNA can control the levels of multiple messenger RNAs to regulate biological processes, such as apoptosis, differentiation and metastasis, and can play a role in tumor development [77]. MicroRNA-125a has been found to be associated with HCC development in chronic HBV infection [5,78].

By comparing the epigenetic changes in normal liver tissue cells and tumor cells of HCC patients, we can reveal the mechanisms of carcinogenesis induction, and find new diagnostic biomarkers and targeted therapeutic targets. *MINPP1* is a tumor suppressor, which can inhibit tumor proliferation and metastasis. A group of researchers used microarray analysis to compare genes and microRNAs in liver tissues of HBV-positive and HBV-negative HCC patients and found that microRNA-30B-5P significantly down-regulated *MINPP1* expression in hepatocytes of HBV-positive HCC patients and promoted the glycolytic bypass pathway, significantly enhancing the proliferation and migration of tumor cells [79]. However, microRNA-30B-5P/*MINPP1* cannot regulate glycolytic bypass to promote tumorigenesis in HBV-negative HCC cells [79]. A further bioinformatic analysis of large cohort data showed that high *MINPP1* expression was associated with better survival in HBV-positive HCC patients, which may contribute to the slower progression of the disease. These results demonstrate the potential value of miRNA-30B-5P/*MINPP1* as a new biomarker for the early diagnosis of HBV-positive HCC and a potential drug target for antitumor therapy [79].

### 2.3. HBV Gene Mutation

HBV is currently divided into nine recognized HBV genotypes (A to I) and one to be determined (J). Genetic diversity and viral mutations cause different HBV infections to have different pathogenic characteristics and lead to different clinical outcomes. Genotype C and D infection are high risk factors for the development of HCC in patients with chronic hepatitis B [5,50]. HBV DNA sequencing results showed that HCC and cirrhosis patients with HBV integration were dominated by HBV genotype C [14]. Mutations in the HBV gene may further increase the carcinogenic risk of infection with specific genotypes of viruses. Several specific HBV variants are known to cause HBV-associated carcinogenesis, including the HBV pre-c mutation G1896A, the pre-s gene deletion, the basal core promoter (BCP) A1762T/G1764A mutation, and the HBx gene K130M + V131I + V5M double or triple mutations [45,49,50,80]. Mutations in the basal core promoter (BCP) region enhance viral replication by creating binding sites for hepatocyte nuclear factor (HNF) proteins, resulting in increased viral RNA transcription or enhanced viral encapsulation [45]. The A1762T/G1764A double mutation is the most common mutation in BCP and has been shown to significantly and independently increase the risk of HCC in patients with chronic HBV genotype C [50]. The G1896A mutation in the pre-core region (PC) can regulate HBeAg synthesis at the translational level by introducing a stop codon [81]. A specific HBV genotype F1b was found to be strongly associated with HCC in Alaska Native young adults [50]. A complete sequence analysis showed a significant increase in T1938C/A2051C mutations in the core region of HBV from this F1b genotype, including A1762T/G1764A mutations in the basal core promoter region (BCP) and G1896A mutations in the pre-core region (PC) [50]. Although the specific mechanism by which F1b genotype HBV induces HCC remains unclear, the accumulation of these core mutations can promote the development of early HCC [50]. 

In addition to mutations in the core region of HBV, mutations in some other regions may also be associated with the occurrence of HCC, which can be used as diagnostic and therapeutic targets for HBV-induced HCC. The HBV RT gene is a target sequence for antiviral therapy with nucleoside analogues, but due to the high replication rate of HBV, immune pressure from the host, and error-prone HBV reverse transcriptase, frequent mutations may occur, leading to the immune escape of HBV and potential carcinogenesis [82]. In a study of 307 patients with chronic hepatitis B (CHB) and 237 patients with HBV-induced HCC, researchers performed high-throughput parallel sequencing of HBV-infected RT genes in these patients and applied machine-learning (ML) algorithms to identify internal quasi-species patterns in the HBV RT region for the prediction of individual HCC. This model is effective in differentiating HCC from CHB and has impressive predictive performance [80]. Hepatitis B virus X (HBx) mutations also increase HCC. Among them, HBx combination mutants and the carboxylic-acid-terminal-truncated HBV X protein (Ct-HBx) are considered to have a higher carcinogenic risk [83,84]. HBV integration into the host genome often results in the truncation of the HBV genome, especially at the C-terminus of hepatitis B virus X (HBx), resulting in the production of Ct-HBx. The most common HBV X gene encoding Ct-HBX in HBV-HCC samples significantly increased the aggressiveness of HCC compared with the full-length X gene [84]. A team of researchers injected wild-type HBx (WT-HBx) and four HBx mutants (M1: A1762T/G1764A; M2: T1674G + T1753C + A1762T/G11764A; M3: C1653T + T1674G + A1762T/G2764A; and Ct-HBx) into mice to observe the association of HBx mutations with HCC. [83]. The results showed that the incidence of HCC was higher in mice injected with M3-HBx and Ct-HBx. By cDNA microarray analysis, M3-HBx and CtHBx significantly up-regulated the expression of plasminogen activator inhibitor-1 (*PAI1*) and cell division cycle 20 (CDC20) in the liver and induced pro-cancer inflammation to promote carcinogenesis [83]. Silencing *PAI1* attenuates the effect of this mechanism on cells. *PAI1* can be an important predictive and prognostic biomarker and a promising therapeutic target for HBV-HCC [83]. Considering the high rate of HBV gene mutation and its close association with HCC, some specific inducers of HBV gene mutation can also be used as HCC-specific biomarkers. APOBEC3G (A3G) cytidine deaminase is an innate immune limiting factor that edits and inhibits hepatitis B virus (HBV) replication. A3G induces specific HBV gene mutations through deamination, which can provide a basis for HCC screening [85].

### 2.4. Progress in Treatment

**Figure 3:**
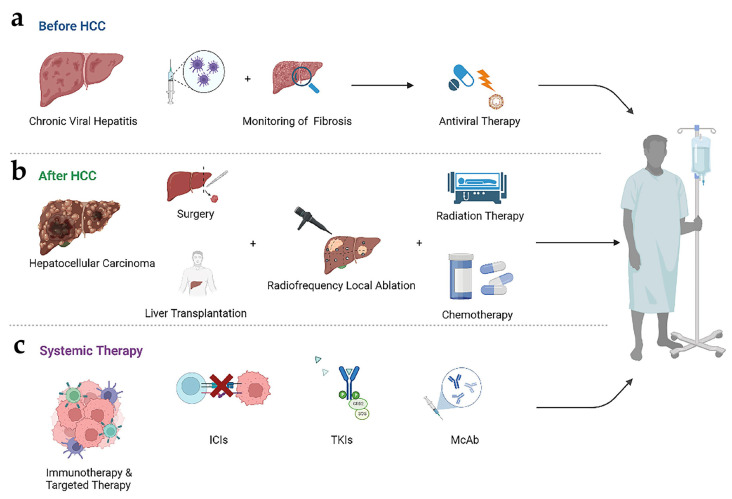
Management and treatment of HBV-induced HCC. (**a**) The main measures before HCC are active HBV vaccination and the continuous detection of the progression of liver fibrosis. For patients with chronic HBV infection, antiviral therapy should be actively carried out to prevent the further development of viral hepatitis. Chronic HBV infection refers to an HBV-positive test, the course of the disease being more than half a year or the onset date not being clear, and clinical manifestations of chronic hepatitis [94]; (**b**) The treatment of HCC includes surgical and non-surgical methods. The surgery includes liver resection and liver transplantation. Non-surgical methods include radiofrequency local ablation, chemotherapy, and radiotherapy [88]; (**c**) Systemic therapies, including immune checkpoint inhibitors (ICIs), tyrosine kinase inhibitors (TKIs), monoclonal antibodies (McAbs) and other targeted molecular drugs, can better achieve personalized treatment, especially to improve the survival rate of advanced patients who cannot be treated with surgery. The image material is referenced from Biorender [2].

Early prophylaxis and treatment are the strategies of choice to reduce the incidence of HBV-induced HCC, including vaccination to prevent HBV infection, the monitoring of hepatic fibrosis progression with reliable biomarkers, and the use of effective antiviral therapies such as nucleo(s/t)ide analogue (NAs), entecavir (ETV), tenofovir alafenamide fumarate (TAF) and tenofovir disoproxil fumarate (TDF) (Figure 3a) [2,5,86,87]. These measures can greatly change the prognosis of patients with chronic HBV infection, thereby improving liver histology, preventing progression into HCC, and improving the survival rate [5]. However, vaccination does not completely prevent HBV infection. The existing antiviral therapy also has difficulty in achieving a complete cure of HBV infection, because these drugs cannot eliminate cccDNA in liver tissue [35]. Every year, many HBV-infected patients still develop CHB, which greatly increases the possibility of HCC. The conventional treatment of HCC includes surgical treatment and non-surgical treatment. Surgical treatment is the main treatment for HCC patients, including hepatectomy and liver transplantation. Chemotherapy and radiotherapy are commonly used for HCC patients who cannot be treated surgically. Radiofrequency local ablation is the main method for the non-surgical treatment of early HCC guided by imaging (Figure 3b) [88]. In terms of drug therapy, HCC is relatively resistant to traditional chemotherapy drugs, such as 5-fluorouracil, cisplatin, doxorubicin or gemcitabine [3]. The emergence of targeted molecular drugs, such as sorafenib, lenvatinib and other second-line medications, has led to better supportive care for patients with advanced HCC who cannot be operated on. Despite the remarkable clinical efficacy of molecular therapies in patients with advanced HCC, drug resistance almost inevitably emerges, hindering eventual cure. Cancer heterogeneity and the misjudgment of clonal drivers are the main reasons for the failure of molecular therapy and the induction of drug resistance [89]. Regional and ethnic differences also lead to significant heterogeneity in these treatments [6]. At present, new molecular mechanisms related to HBV-induced HCC disease have been continuously discovered, which also creates more potential for the development of new targeted therapies. Given that HBV is a virus with a reverse transcriptase replication step, some of the mechanisms used to inhibit infection with retroviruses such as HIV may also be effective. *SLFN11*, a member of the human Schlafen family, has previously been shown to inhibit the production of human immunodeficiency virus 1 (HIV-1) through codon usage [90]. Recent studies have found that *SLFN11* expression is decreased in HCC, suggesting that *SLFN11* may play a role in inhibiting HBV-induced HCC tumor progression. A further mechanism analysis revealed that *SLFN11* may inhibit the mTOR signaling pathway through *RPS4X*, while the mTOR pathway inhibitor INK128 could achieve a similar tumor suppression effect. This study suggests that INK128 and *SLFN11* can be used as new therapeutic strategies for HCC [91]. Similarly, some targeted drugs used in the treatment of other tumors may also be effective in HCC. Recent studies have found that the use of the *SOAT1* inhibitor AvasimiBE can inhibit the proliferation and migration of HCC cells, and high levels of *SOAT1* expression have previously been shown to be associated with a poor prognosis in prostate and pancreatic cancer [92].

The HBV-induced persistent host immune response and abnormal regulation of the immune system play a very important role in the pathogenesis of HBV-induced HCC [8]. Immunotherapy for HCC including checkpoint inhibitors and monoclonal antibodies is one of the research hotspots. Some immune checkpoint inhibitors, such as Nivolumab (anti-PD-1 mab), have been approved for the treatment of advanced HCC patients treated with sorafenib in several countries and regions, and have shown reliable safety [6]. Recent studies have demonstrated that HBV contains α2,6-biantennary sialoglycans that can regulate the host immune response by binding to sialic-acid-binding IgG-like lectins (SIGLECs), which may contribute to HBsAg-induced immunosuppression. Based on this result, researchers found that the combination of anti-SIGLEC-3 mab and GS-9620 could activate host immunity to produce anti-HBsAg antibodies, clear HBsAg, and reduce the incidence of HCC in CHB patients [93].

Compared with traditional HCC treatment methods, the use of systemic therapies, including immune checkpoint inhibitors (ICIs), tyrosine kinase inhibitors (TKIs), monoclonal antibodies and other targeted molecular drugs can better achieve personalized treatment, especially to improve the survival rate of advanced patients who cannot be treated with surgery (Figure 3c) [2]. The continuous exploration of the disease mechanism of HBV-related HCC is crucial for us to enrich therapeutic methods and deal with tumor resistance.

## 3. Hepatitis-C-Virus-Induced Hepatocellular Carcinoma

Hepatitis C virus (HCV) infection accounts for approximately 30% of HCC cases, affecting more than 71 million people worldwide [4,8,19,95]. HCV is a hepatophilic, positive-strand RNA virus in the Flaviviridae family. HCV is divided into seven genotypes and different subtypes, and the distribution of genotypes varies among different populations, with types 1, 2 and 3 dominating overall [19,96,97]. In contrast to HBV, HCV does not integrate its viral genes into the host cell genome and therefore does not cause direct mutations in host cell genes. HCV-induced HCC is mainly related to the persistent host immune response and chronic inflammation caused by the failure of acute infection clearance, which further leads to the occurrence of liver fibrosis and cirrhosis, and eventually develops into HCC [4,8]. In general, the adaptive immune response associated with HCV is delayed, weak, and ineffective, and this immune dysfunction makes most HCV infections chronic [98]. Long-term immune response and inflammation can cause continuous damage to liver tissue and stimulate the occurrence of liver fibrosis. Liver fibrosis is a key step in the development of HCV-induced HCC, which can further lead to the occurrence of cirrhosis [98]. Most associated HCCs are based on the background of liver fibrosis or cirrhosis. Even after HCV clearance, residual liver fibrosis still has the risk of progression to HCC, which requires additional monitoring and treatment [19].

Approximately 130–170 million people worldwide have chronic HCV infection, and these patients are at high risk of progression to HCC and require early and effective antiviral therapy [99,100]. Treatment that induces a sustained viral response (SVR) is a reliable marker of HCV eradication and is associated with the inhibition of liver disease progression and the associated complications, including HCC development [101]. Thanks to the use of direct-acting antiviral (DAA) therapy, an increasing number of patients with HCV infection are successfully treated to SVR, which has greatly reduced the risk of HCC (50–80%) [2,5,101]. However, several studies have reported that a sustained virological response (SVR) does not eliminate the development of cancer, and HCV patients still have the possibility of relapse after DAA treatment. In a study of HCV incidence trends in the U.S. Veterans Health Administration (VHA) from 2002 to 2018, antiviral therapy did not completely eliminate residual HCC risk, especially in patients with advanced fibrosis, although extensive HCV treatment decreased HCC risk [102]. Both viral and host-related factors appear to influence HCC development after HCV RNA eradication. A study of the incidence of liver cancer in 1922 patients with HCV genotype 1 or 2 (HCV-1 or HCV-2) associated with chronic liver disease after DAA treatment to SVR showed an association between the HCV-1B core amino acid (aa) 70 mutation (virus-related factor) and the development of HCC. Meanwhile, alpha-fetoprotein (host-related factor) can be used as a predictor of HCC after HCV treatment [103]. A prospective multicenter study from Italy further evaluated the incidence of early HCC and its risk factors in an HCV-infected population treated with DAAs. The researchers followed 985 patients treated with DAAs for 48 weeks. A total of 35 HCC patients were observed during the follow-up. A multivariate analysis indicated that sofosbuvir (without ribavirin) therapy was independently associated with HCC development. Ribavirin appears to play a protective role in HCC development [104]. Other studies have shown that DAA-induced SVR has no effect on the development of short- and medium-term HCC but can reduce the risk of developing medium- and long-term HCC [105]. The role of DAAs in the incidence or recurrence of hepatocellular carcinoma (HCC) in patients with HCV remains controversial. Different treatments and different periods can show different results. In addition, recent clinical observations and further cohort studies indicate that DAA treatment appears to cause HBV reactivation (this will be mentioned later) [106]. This also makes the risk of HCC development in patients treated with DAAs more unclear.

Further studies are needed to understand the molecular mechanisms underlying the risk of HCC progression after the HCV cure, but different HCV genotypes, subtypes, and gene mutations seem to play a role [100,103]. The recurrence of HCV-induced HCC is not only manifested in the progression to HCC after the cure of HCV infection, but also in the recurrence of HCC patients after surgical resection. Studies have shown that HCV-associated HCC shows more aggressive tumor characteristics, higher tumor multifocal nature and vascular infiltration, leading to an increased recurrence rate after therapeutic resection [4]. Using a functional and genomic screening system, researchers found that HCV infection activates epidermal growth factor receptor (EGFR) signaling, which promotes invadopodia formation and function. Through this mechanism, HCV infection leads to a higher aggressiveness of HCC tumors, resulting in a worse prognosis and higher recurrence rate [4]. Although the relative risk of HCV progression to HCC and HCV recurrence after DAA treatment remains controversial, the absolute risk of these events cannot be denied. This poses a challenge for the further optimization of HCV-induced HCC treatment measures.

Another concern is HBV and HCV co-infection. The disease progresses more rapidly in individuals with HBV/HCV co-infection than in individuals with a single infection. Patients with chronic HBV/HCV co-infection have faster fibrosis progression and a greater risk of cirrhosis, liver decompensation, and HCC than patients with a simple infection with either virus [107,108,109]. At present, the global prevalence of HBV/HCV co-infection is unclear and varies in different regions. However, in geographic areas where both infections are prevalent, HBV/HCV co-infection is more common due to the common transmission route of the two viruses [110]. In a cohort study of 2172 histologically confirmed PLC patients from the National Cancer Center/Cancer Hospital of the Chinese Academy of Medical Sciences, 1823 (83.9%) were HCC. The positive rate of HBV and HCV co-infection markers in these HCC patients was 6.7% (122/1823) [111]. In addition, HBV/HCV co-infection is common in people living with HIV (PLHIV). In China, a study included 6611 cases (*n* = 1571 cases in 2007; *n* = 5040) cohort study of HIV-infected persons in 2015 showed that the prevalence of HBV/HCV co-infection in PLHIV was 61.1% in 2007 and 18.0% in 2015, respectively [112]. This number decreased to 2.7% (*n* = 1984) in another study in 2017 [113].

Recently, the use of DAAs to treat HBV reactivation in patients with HCV has become an important clinical consideration and has further attracted attention to HBV/HCV co-infection [106]. HBV reactivation, generally considered to be a sudden increase in HBV replication in patients with inactive or resolved HBV infection, may lead to clinically significant hepatitis [110]. A laboratory diagnosis is manifested by the redetection of HBV DNA in individuals with no previously detected HBV DNA or an increase in serum HBV DNA levels of 1 to 2 log IU/mL. HBsAg serum reversal in HBcAb-positive, HBsAg-negative patients can also be recognized as HBV reactivation [106]. Typically, HBV reactivation is associated with an immunosuppressive status in patients, and DAA therapy does not have such an effect. However, a growing body of research supports an association between DAA treatment and HBV reactivation. In a cohort of 111 HCV-infected patients, HBV reactivation during ledipasvir/sofosbuvir treatment and 108 weeks of follow-up were evaluated. HBV virological reactivation occurred in 73% of patients (81/111). Clinical reactivation occurred in 9% (10/111). Most HBV virological reactivation (86%, 70/81) occurred at week 12 of follow-up, and clinical reactivation was usually more delayed. HBV clinical reactivation was defined as HBV reactivation with alanine aminotransferase (ALT) > 2 × upper limit of normal (ULN; 43 U/L in men and 34 U/L in women) at the same time [108]. It can be seen that DAA treatment promotes HBV reactivation to some extent. However, this reactivation does not generally seem to have serious consequences. From 22 November 2013 to 15 October 2016, the FDA identified 29 reports of HBV reactivation in patients receiving DAAs, with only 3 cases of decompensated liver failure, 2 of which resulted in death and 1 requiring liver transplantation [110]. In another systematic review and meta-analysis, the HBV reactivation risk in 1621 patients with chronic HBV infection (*n* = 242) or resolved HBV infection (*n* = 1379) treated with different DAAs was 24% (chronic HBV infection) and 1.4% (resolved HBV infection), respectively. Only three major clinical events related to HBV reactivation were reported in patients with chronic HBV infection (one case of liver decompensation and two cases of liver failure, one of which required liver transplantation) [114].

At present, the mechanism of HBV reactivation caused by DAA treatment remains unclear. Because DAA treatment works by inhibiting the proteins required for HCV replication, it does not cause host immunosuppression. One of the popular theories is the viral interference theory, which states that active HCV replication creates a host immune state that is conducive to controlling HBV replication, and that DAA treatment disrupts this immune state. HCV infection is thought to inhibit HBV replication in patients with HBV/HCV co-infection and is mediated by several HCV proteins, including core, NS2, and NS5A. Therefore, the DAA inhibition of HCV may lead to increased HBV replication and protein expression [106,108,110,114]. However, other studies have refuted the viral interference theory by proving that HCV and HBV can replicate in the same liver cells [115]. Therefore, the molecular mechanism involved in HBV–HCV interference is still controversial. As to whether HBV reactivation due to DAA therapy increases the risk of HCC development, there is insufficient evidence. This is a reasonable concern given the large number of people infected with HBV and HCV. However, this situation may not be of immediate concern to physicians, and DAA treatment appears to be safe in patients co-infected with HBV and HCV. However, given the high rate of HBV reactivation, we still recommend antiviral prophylaxis in patients with chronic HBV and HCV co-infection.

## 4. Hepatitis-D-Virus-Induced Hepatocellular Carcinoma

Hepatitis delta virus (HDV) is a circular single-stranded negatives-sense RNA-deficient virus that encodes only one δ protein or δ antigen (HDAg) and must be dependent on the presence of the HBV surface antigen to replicate, thus affecting only HBV-infected patients [2,22]. Hepatitis D affects an estimated 20 to 40 million people worldwide, and approximately 5% of HBV-infected people worldwide are co-infected with HDV. Chronic infection with HDV is considered the most severe form of viral hepatitis infection in humans. HDV infection progresses more rapidly than other chronic viral hepatitis infections, is more likely to lead to cirrhosis, and is associated with an increased risk of HCC [21,116,117]. HDV infection can occur through HBV co-infection (co-infection with both viruses during the same exposure) or superinfection (infection with HDV after an established HBV infection, such as in HBsAg-positive individuals). Chronic HDV infection is more serious than chronic HBV infection. The progression rate of liver fibrosis is increased in patients with co-infection, and the risk of HCC is three times higher than that in patients with a single HBV infection [21].

As for the mechanism of HDV-induced HCC, the results of the current studies are not very clear. Because HDV is an RNA virus that cannot integrate viral genes into the host genome and must be dependent on HBV for replication, it is unlikely that HDV directly causes HCC. HDV-induced HCC is often associated with the interaction between HDV and HBV, and aggravates the development of fibrosis and cirrhosis in chronic viral hepatitis [22]. HDAg seems to play a role in hepatocyte transformation. Studies have shown that L-HDAg may promote inflammation through the activation of signal transducers and activators of transcription (STAT-3) or the activation of NF-κB induced by oxidative stress. The effects of this inflammatory response include ER stress and necrotizing inflammation, as well as a possible increase in reactive oxygen species production, which may ultimately contribute to HCC development [21,22,116]. In addition, some HDV-induced epigenetic changes (such as lncRNAs) may also be involved in the process of hepatocyte transformation, and the mechanism needs to be further elucidated.

In terms of treatment, the poor efficacy of HBV-specific nucleoside analogues in the treatment of HBV and HDV co-infection, as well as the specificity of the HDV life cycle, hinder the development of HDV-specific drugs. At present, the only clinical treatment for HDV is IFN-α. However, it is difficult to achieve a long-term virological response and cannot be used in patients with cirrhosis, active autoimmune diseases or some psychiatric disorders [116]. Therefore, it is necessary to design more effective and safe antiviral drugs for patients with HBV complicated with HDV infection. As our understanding of the HDV life cycle has advanced, drugs targeting more mechanisms of HDV infection have been proposed, including more specific interferon α, viral entry inhibitors, HBsAg secretion inhibitors, and viral assembly and packaging inhibitors. These drugs create the possibility of more personalized and effective treatment in the future [21,116]. Among these drugs, lonafarnib acts as an entry inhibitor to specifically treat HDV infection. HDV assembly requires the post-translational farnesylation of L-HDAg by cellular farnesyltransferase. Lonafarnib blocks the HDV virus assembly process in vivo by inhibiting farnesyltransferase. Therefore, lonafarnib can directly interfere with the release of HDV particles from infected liver cells to play a therapeutic role [116]. Lonafarnib’s ability to inhibit HDV was demonstrated in clinical trials, where results showed that treatment with lonafarnib for chronic HDV significantly reduced viral levels. The decrease in virus level was significantly correlated with serum drug level [118]. However, it is worth noting that lonafarnib inactivates an essential cell enzyme (farnesyltransferase) and may affect normal cell life, including the farnesylation of signaling molecules such as c-Ras. Therefore, long-term use of lonafarnib in the treatment of HDV infection should be concerned about the possible adverse reactions [116]. In addition, recent studies have demonstrated that specific nucleic acid polymers (NAPs) can also inhibit HDV. For example, a highly negatively charged nucleic acid polymer based on intravenous administration can interfere with HBV and HDV attachment to heparan sulfate proteoglycans (HSPGs) [116,119], thus inhibiting the binding of HBV and HDV to specific NTCP. In addition, some related nucleic acid polymers have also been shown to inhibit the secretion of HDV virions, because the secretion mechanism of HDV virions is similar to that of HBV subvirions. In a clinical study, results showed that treatment in combination with REP 2139 (a nucleic acid polymer) and pegylated interferon alpha-2a was safe and well tolerated, and established functional control of HBV and HDV co-infection and the normalization of a high proportion of serum transaminases one year after treatment [120].

## 5. Hepatitis E Virus and Hepatocellular Carcinoma

Hepatitis E virus (HEV) is a positive single-stranded RNA virus belonging to the genus Orthohevirus A of the Hepeviridae family. Its genome is composed of 7.2 kb ssRNA. The virions are icosahedral in shape and 27–32 nm in diameter. HEV is a “quasi-enveloped” virus that exists in both non-enveloped and enveloped (“eHEV”) forms. The virus mainly infects liver cells and causes human hepatitis E [121,122,123]. As a highly infectious non-enveloped virus, HEV is mainly transmitted through the fecal–oral route, and the particles secreted in the blood or produced in cell culture are coated with lipid membranes from the host cells. This lipid membrane protects the virus from being neutralized by antibodies and plays an important role in cell tropism, but it also reduces the infectivity of the virus. Both naked HEV particles and quasi-coated HEV particles have strong resistance to alcohol-based disinfectants [123,124,125]. Hepatitis E virus (HEV) infection can cause acute and chronic hepatitis, as well as some extrahepatic damage, and is one of the most common causes of acute viral hepatitis worldwide. More than 20 million people are infected with HEV each year, and about 50,000–70,000 people die from hepatitis E virus infection [126,127,128]. Four main HEV genotypes cause human disease: types 1, 2, 3, and 4. Types 1 and 2 of HEV infect humans, while type 3 and type 4 of HEV are zoonotic viruses [33]. Although HEV affects a large number of people, it generally causes only mild symptoms such as fever, fatigue, vomiting, and jaundice in most patients. Hepatitis E infection is usually self-limiting and resolves in two to six weeks. [34]. The acute infection mortality rate of HEV infection is usually about 2% or even lower [129]. It is important to note that HEV 1 and 2 can cause severe explosive hepatitis in pregnant women and increase the risk of liver failure, while increasing the mortality rate to 20–25% [34,130].

At present, there is still no consensus on whether HEV infection leads to HCC, which is largely related to the fact that HEV generally only leads to acute infection. However, one case in 2008 described an unexpected finding of chronic hepatitis E infection (persistent viremia over 3–6 months and excluding HBV co-infection) in organ transplant recipients, and chronic persistent HEV infection in immunodeficient patients has been reported in subsequent years [31,32]. This has led to new efforts to study the pathogenesis of chronic hepatitis E virus infection and identify new treatments. It is now known that HEV infection has the potential to develop into a chronic infection in immunodeficient patients, such as cancer patients receiving chemotherapy and/or immunotherapy and autoimmune disease patients receiving immunosuppressive or immunomodulatory therapy [122]. HEV antibodies are often undetectable in these patients and need to be diagnosed by nucleic acid amplification. According to large cohort studies and meta-analyses in European countries, HEV infection became chronic in about two-thirds of solid organ transplant recipients [131,132]. HEV can cause chronic infection, which means that it has the same potential to cause HCC as other hepatitis viruses (HBV and HCV) that cause chronic infection. Some mechanisms of chronic viral hepatitis leading to HCC are common, such as long-term chronic inflammatory infiltration and disruption of cellular pathways caused by the interaction between viral proteins and host factors [133,134]. It is particularly noteworthy that a case of HCC complicated with chronic hepatitis E was described in a report, suggesting that HEV is closely related to the development of HCC, if not the primary cause of HCC [30].

However, the link between HEV and HCC remains uncertain, especially the effects of immunodeficiency and HBV co-infection. There have been a number of studies worldwide focusing on the link between HEV and HCC. In China, researchers recruited 950 cancer patients and 950 control volunteers from East China’s Shandong Province to participate in a survey of HEV seropositivity in tumor patients. The positive rate of HEV in 103 liver cancer patients was found to be three times higher than that in 950 control volunteers (31% vs. 13%) [135]. Similarly, investigators in Cameroon found that HCC patients showed a higher seroprevalence of HEV infection, dominated by IgG HEV antibodies, suggesting a more persistent infection. Compared with non-HCC subjects with chronic liver disease, more anti-HEV IgG was observed in HCC patients (41.8% vs. 12.6%) [136]. In addition, HCC patients with current or past HEV infection had increased liver enzyme levels and prothrombin time but decreased platelet counts compared with HCC patients without HEV infection. This observation suggests that HEV infection contributes to worsening liver inflammation and promoting the carcinogenesis process [33,136]. In fact, many HEV-infection-associated HCC patients are co-infected with HBV, which may affect the role of HEV in HCC development. On the one hand, a co-infection of HEV and HBV may accelerate the development of HCC (including the synergistic effect of HBV with HEV and the acceleration of the disease course of HBV chronic infection by HEV). On the other hand, a co-infection of HEV and HBV has been suggested by some researchers to have antagonistic effects (similar to competition) that may reduce the risk of CHB developing into HCC. In Taiwan, China, researchers conducted a study to determine whether repeated HEV infection in CHB patients is associated with an increased incidence of liver-related death, cirrhosis, and HCC. Although the study did not show a direct link between repeated HEV infection and HCC development, repeated HEV infection was considered as a risk factor for HCC development in 723 HBeAg-negative patients with chronic HBV infection [34]. In addition, the one-year mortality rate of HBV-associated cirrhosis in HEV patients was 35.7%. This suggests that HEV may be a risk factor for accelerating the progression of CHB disease [34]. A study in Vietnam suggested that HBV-infected patients had a higher serum-positive rate of HEV than the control group (IgG: 45% vs. 31%; IgM: 11.6% vs. 4.7%). Serologically positive HEV was found in nearly half of liver cancer or HCC patients [33]. Similar to those mentioned above, repeated HEV infection is associated with abnormally increased levels of bilirubin, albumin, and prothrombin, suggesting that repeated HEV infection aggravates the severity of HBV-induced chronic inflammation [33]. Although many studies have shown a catalytic role of overlapping HEV infection in disease progression and transformation to HCC in CHB patients, some studies have shown different results. In a case-control study of a population in southern China, HEV infection did not aggravate and may have even reduced the promoting effect of HBV on HCC development. The results showed that the risk effect of HEV infection on HCC was not statistically significant (OR: 1.22, 95%CI: 0.68–2.19), and there was no significant proliferative interaction between HEV and HBV infection on HCC risk (OR: 0.80, 95%CI: 0.36–1.79) [137]. These different results make the relationship between HEV and HCC more complicated. This complex result is influenced by a number of factors: First, chronic HEV infection appears to be limited to severely immunocompromised individuals, and therefore its overall incidence is low, making it difficult to obtain study subjects. Second, HEV may only be a co-factor in HCC development, and its role in HCC is easily overridden by other pathogens such as HBV, HCV, and/or alcohol. In addition, when chronic HEV infection is confirmed, about 80% of patients can be cured by reducing the dose of immunosuppressive drugs or by ribavirin for three months. Almost all cases that are virologically unresponsive or relapsed can be cured by an additional six-month course. All these may have contributed to the neglect of HEV infection and its role in HCC patients [134].

Although some studies have discussed whether HEV is related to HCC, these results are obviously insufficient and cannot directly prove that HEV is a factor in HCC occurrence. Larger case studies and a deeper exploration of mechanisms are needed. However, the available results demonstrate the value of monitoring HEV infection status, including serological monitoring and HEV RNA detection, in immunocompromised and CHB patients.

## 6. Conclusions

HCC is the fifth most common cancer and the second most common cause of cancer death in the world. Its incidence and mortality are still increasing, and it has become an important public health problem. Among all the risk factors for HCC, infectious factors, including HBV, HCV and HDV, are the most important. This is not only because these infectious factors play a major role in the pathogenesis of HCC, but also because there are a large number of patients with chronic viral hepatitis worldwide, who are at high risk of developing HCC (Table 1). HBV infection is the most serious infectious factor of HCC, especially in Asia. The mechanisms of HBV-induced HCC include viral DNA integration, epigenetic changes, immune dysfunction and viral gene mutations. Many new diagnostic and therapeutic approaches have been explored to address these mechanisms. However, the current treatments do not eliminate cccDNA. For HBV-infected individuals, the risk of progression to CHB and HCC is always present. Therefore, further research will focus on the early diagnosis and treatment of HBV and how to achieve a complete cure. An increasing number of patients have achieved SVR thanks to the use of DAAs for HCV, but the absolute risk of HCC and HCV recurrence in HCV patients after treatment remains a concern. Co-infection of HDV and HBV will greatly increase the possibility of HCC development, leading to a poor prognosis. However, at present, the mechanism of HDV involvement in HCC has not been clarified, and drugs targeting HDV are also scarce, so more research investment is urgently needed. Targeted therapy and multi-system combination therapy are important treatment modes for HCC in the future, which require more in-depth and comprehensive studies on the pathogenesis and infectious factors in different populations in different regions. The development of effective treatment modalities can improve the prospects of survival and quality of life in patients with HCC at all stages. In addition, more studies on the association between HEV and HCC are needed, including larger-scale case studies and more in-depth mechanism studies. However, given the increasing recognition of chronic HEV infection, it is reasonable to speculate that HEV plays a role in the development and progression of HCC. This concern has further led to new considerations regarding the need for HEV testing in HCC patients and the need for the prevention of HCC in HEV-infected patients. Although, with the continuous optimization of health management and preventive treatment measures, the role of hepatitis virus as a risk factor and cause of HCC is no longer the same as before. However, it cannot be denied that hepatitis virus, represented by HBV, still plays an important role in the occurrence and development of HCC with its large infected population and complex pathogenic mechanism, which seriously threatens people’s health. However, due to the medical pressure brought about by the COVID-19 pandemic, the diagnosis and treatment of hepatitis virus are becoming more and more challenging, which tests the planning and coordination of policymakers in various countries and calls for more investment in related research.

In this review, we mainly summarize recent studies on the association between hepatitis virus and HCC and discuss the implications of these findings for the management of infection-associated HCC. Of course, there are still limitations in this paper, including possible omissions of research findings and the failure to fully discuss the current diagnosis and treatment of HCC. However, we believe that these views can provide inspiration and reference for the study of HCC and hepatitis virus and call for the optimization of HCC management policies related to hepatitis virus.

## Figures and Tables

**Table 1:** Overview of the mechanisms of hepatitis virus induction into HCC.

Hepatitis Virus	Mechanisms	Type	References
HBV	HBV integrations promote local and distant oncogenic driver alterations (TERT, TP53, MYC) in HCC *	DNA integration	[17]
	HBV integrations in multiple cancer-associated genes (such as TERT, MLL4, and CCNE1) *	DNA integration	[26]
	HBV-TERT promoter integration harnesses host ELF4 resulting in TERT gene transcription in HCC *	DNA integration	[42]
	HBV-induced increased N6 methyladenosine modification of PTEN RNA affects innate immunity and contributes to HCC *	Epigenetic Changes	[72]
	Hepatitis B protein HBx binds the DLEU2 lncRNA to sustain cccDNA and host cancer-related gene transcription *	Epigenetic Changes	[35]
	HBV P protein initiates glycolytic bypass in HCC via a FOXO3/miRNA-30b-5p/MINPP1 axis *	Epigenetic Changes	[79]
	Core mutations in HBV genotype F1b	Gene Mutation	[50]
	HBV reverse transcriptase gene mutation	Gene Mutation	[80]
	HBV X gene mutants	Gene Mutation	[83]
	A3G-induced HBV DNA mutations *	Gene Mutation	[85]
	Low expression of SLFN11 in HCC *	Abnormal expression of specific protein	[91]
	High expression of SOAT1*	Abnormal expression of specific protein	[92]
HCV	Chronic inflammatory response and hepatic fibrosis	Immune and inflammatory response	[98]
	DAA therapy induces HBV reactivation and HCC development (to be further demonstrated) *	Therapeutic influence	[104,106]
HDV	Interactions between HDV and HBV and the development of chronic viral hepatitis fibrosis and cirrhosis	HBV co-infection	[22]
	Activation of STAT-3 or oxidative-stress-induced activation of NF-κB promotes inflammatory reaction *	Inflammatory reaction	[116]

* TERT: telomerase reverse transcriptase; TP53: tumor protein p53; MLL4: mixed lineage leukemia 4; CCNE1: cyclin E1; ELF4: E74-like ETS transcription factor 4; PTEN: phosphatase and tensin homolog; DLEU2: deleted in lymphocytic leukemia 2; FOXO3: forkhead box O3; MINPP1: multiple inositol-polyphosphate phosphatase 1; A3G: APOBEC3G; SLFN11: Schlafen family member 11; SOAT1. sterol O-acyltransferase 1; DAAs: direct-acting antiviral agents; STAT-3: signal transducers and transcriptional activators 3.
